# A health-social service partnership programme for improving the health self-management of community-dwelling older adults: a hybrid effectiveness-implementation pilot study protocol

**DOI:** 10.1186/s40814-023-01412-0

**Published:** 2023-11-08

**Authors:** Arkers Kwan Ching Wong, Frances Kam Yuet Wong, Karen Kit Sum Chow, Dilys Kwai Sin Kwan, Dubby Yun Sang Lau, Avis Cheuk Ki Lau

**Affiliations:** 1 School of Nursing, The Hong Kong Polytechnic University, 1 Cheong Wan Road, Hung Hom, Hong Kong; 2 The Hong Kong Lutheran Social Service, Homantin, Hong Kong

**Keywords:** Health-social service partnership, Self-care, Implementation science, Hybrid effectiveness-implementation, Ageing

## Abstract

**Background:**

The ageing population requires seamless, integrated health and social care services in the community to promote the health of older adults. However, inadequate financial resources, a lack of clear operational guidelines, and various organisational work cultures may affect the implementation quality and sustainability of these services. As a unique approach, this study seeks to examine the preliminary effects of a health-social partnership programme on the health self-management of community-dwelling older adults in Hong Kong. Additionally, the study seeks to ascertain key insights into the mechanisms and processes required to implement and sustain a self-care management programme in broader practice in community settings.

**Methods:**

This study will use a hybrid effectiveness-implementation design. During the 3-month programme, subjects in the intervention group will receive four Zoom video conference sessions and four telephone calls conducted by a health-social service team that will include a nurse case manager, community workers, general practitioners, a Chinese medicine practitioner, and social workers. Subjects in the control group will receive a monthly social telephone call from a trained research assistant to rule out the possible social effect of the intervention. The reach, effectiveness, adoption, implementation, and maintenance framework (i.e. RE-AIM framework) will be used to evaluate the implementation and effectiveness outcomes. Of the five dimensions included in the RE-AIM framework, only effectiveness and maintenance outcomes will be collected from both the intervention and control groups. The outcomes of the other three dimensions—reach, adoption, and implementation—will only be collected from subjects in the intervention group. Data will be collected pre-intervention, immediately post-intervention, and 3 months after the intervention is completed to evaluate the maintenance effect of the programme.

**Discussion:**

This programme will aim to enhance health-promoting self-care management behaviours in older adults dwelling in the community. This will be the first study in Hong Kong to use the hybrid effectiveness-implementation design and involve key stakeholders in the evaluation and implementation of a health self-management programme using a health-social service partnership approach. The programme, which will be rooted in the community, may be used as a model, if proven successful, for similar types of services.

**Trial registration:**

Clinicaltrials.gov, NCT04442867. Submitted 19 June 2020

**Supplementary Information:**

The online version contains supplementary material available at 10.1186/s40814-023-01412-0.

## Background

The rapid increase in life expectancy is outpacing the increase in healthy life expectancy, implying that people are living their extended years with multiple morbidities and complications, physical and cognitive impairment, and frailty [1]. Such complex conditions often lead to challenges with self-care (that is the ability to establish behaviours to promote and maintain one’s health and well-being) for older adults along the health and illness trajectory. In conjunction with knowledge deficits and inadequate social support, self-care activities become increasingly laborious for these individuals [2]. Despite these challenges, self-care activities, including health maintenance, illness prevention, and monitoring and managing healthcare activities, are performed by the older adults and their primary caregivers with limited assistance from healthcare professionals [3]. Increasing awareness and efforts have been devoted to supporting and empowering older adults to perform self-care activities. Evidence shows that this support is pivotal to maintaining, restoring, and improving health and well-being; reducing morbidity, mortality, and healthcare service utilisation rates and the associated costs; and enabling elderly individuals to live independently in the community [4].

As a result of the complicated conditions experienced by older adults, the level of demand for care changes continuously, from an emphasis on providing care in silos to offering interprofessional care [5]. Although there is a comprehensive range of health and social services provided in developed countries [6], the integration of health and social care is still nascent [7]. Many older adults regard health and social services as arduous to comprehend, necessitating the assistance of healthcare professionals to ensure service continuity [5, 6]. A coordinated health and social service partnership may deliver more consistent, continuous, appropriate, and timely services to older adults, and thus achieve favourable health outcomes [8–12]. By minimising redundancies and inconsistencies, services may become more accessible and efficient for older adults with chronic diseases, who require care from different disciplines [8–12].

To address the complex needs of older adults and the fragmentation of health and social care provision, our research team previously implemented a nurse-led, health-social service partnership programme (HSPP) that included proactive comprehensive care assessment, goal setting, self-care self-efficacy enhancement, and coordinated health and social services for a group of community-dwelling older adults [9]. The programme was found to be effective at improving self-efficacy, medication adherence, and quality of life and reducing the utilisation of health services [13]. Other randomised controlled trials using health and social care approaches to address the self-care challenges of older adults with low income and frailty have also reported positive effects on general health and the ability to perform activities of daily living (ADLs) and instrumental ADLs, and reduced hospitalisation rates [14, 15].

Although an increasing number of studies show that a coordinated health-social service partnership approach is useful to facilitate self-care amongst older adults, these studies have used a randomised controlled research design and have been tightly controlled for confounding variables [16]. When implementing a programme in a real-world setting, factors such as heterogeneous samples and settings, attitudes, relationship and organisational cultures of the service providers, and resources and costs may affect the successful adoption and sustainability of the programme [17]. The adaptive process of an intervention in a real-world setting is dynamic and often generates unpredictable issues that require an immediate response from those implementing the programme [17]. A clear conceptualisation and description of the intervention should be delivered to those delivering the programme to ensure that they are capable of adapting the intervention to their own care settings [18]. The degree of constraint at the time of implementation should be determined to allow the effective core components to be maintained under tolerable alterations, and thus, allow the successful adaptation of the intervention with sustained fidelity [19]. Implementation science focuses on the adoption of interventions by systems of care under real-world conditions [20]. All aspects of implementation, including factors affecting implementation, such as the implementation processes and outcomes, generalisation, and continuity, are investigated, with context and users as important components [21]. In a conventional effectiveness study, the barriers to implementation fidelity are often unclear, resulting in frustration and failure to maintain the effectiveness of the programme at the level demonstrated in efficacy trials [22].

To overcome the potential difficulties in translating effective intervention programmes from research to practice, our team aims to conduct a pilot study using a type 1 hybrid effectiveness-implementation design. We used the definition of pilot study that proposed by Eldridge and colleagues [23]. They suggested that pilot study is a subset of feasibility studies that specifically examines a design feature proposed for the main trial, but on a smaller scale. With balanced foci on effectiveness and implementation, the type 1 hybrid design provides an authentic examination of the effectiveness of a programme, while accelerating the translation process [22]. This design also incorporates the shared vision of different stakeholders early in the process of programme development to dynamically address the barriers to and facilitators of implementation, while complementing the direction of the research with system, provider, and client needs [24]. Similar hybrid studies and other implementation studies have addressed the self-care challenges of community-dwelling older adults, but these studies have only focused on either the health or social service domains individually [25, 26]. By gathering information about both effectiveness and implementation strategies, this study aims to provide key insights into the mechanisms and processes required to implement and sustain a self-care management programme in broader practice in community settings.

### Objectives

To evaluate the process of implementing a nurse-led HSPP programme in a community centre, which will include the aspects of the following: *Reaching* into the target population, potential *Effectiveness* of the programme when compared to a monthly social telephone call, *Adoption* by the staff and participants, *Implementation* fidelity, and *Maintenance* over time. Specifically, these aspects are defined as follows:

i) Reach: recruitment rate of the programme.
ii) Potential effectiveness: self-efficacy, quality of life (QoL), health service utilisation (i.e. the total number of unscheduled general out-patient department, general practitioner, and emergency room visits and hospital admissions and the total number of attendances at health services).
iii) Adoption: perceptions on the facilitating factors of and barriers to program adoption in the community centre.
iv) Implementation: fidelity of the programme.
v) Maintenance: sustained programme effects at 6 months.

### Conceptual framework

The hybrid effectiveness-implementation design of this study will be guided by several theories and frameworks. The design of the HSPP intervention will be informed by Bronfenbrenner’s ecological systems theory [27], which asserts that self-care is influenced by factors at the following three levels of the environment: the microsystem, mesosystem, and macrosystem [27]. The microsystem level consists of modifiable personal factors, such as self-efficacy. The mesosystem level focuses on the interrelationships between older adults and the persons who have close connections with them, such as their healthcare providers. The macrosystem level is an extension of the mesosystem level and involves cross-boundary relationships between the different organisations. Based on this theory, the self-efficacy of an individual at the microsystem level may be enhanced when a health-social care service partnership structure is formed at the macrosystem level, with support from healthcare providers at the mesosystem level. Improvements in self-efficacy may promote the self-care management of an individual in the community [28].

The implementation process of the study will be guided by the implementation framework described by Durlak and DuPre [29]. This framework emphasises the following five key components that are required in implementation research: (1) innovation characteristics, such as adaptability and compatibility; (2) provider characteristics, such as self-efficacy and skill proficiency; (3) community factors, such as funding allocation and policy directives; (4) factors associated with the intervention delivery system, such as staffing considerations and organisational factors; and (5) factors associated with the intervention support system, such as staff training sessions and technical assistance. The study will incorporate these components in the implementation process.

The reach, effectiveness, adoption, implementation, and maintenance (RE-AIM) framework will be used to guide the evaluation of the effectiveness and implementation outcomes [30]. The RE-AIM framework was considered for inclusion as it is helpful in ascertaining not only the impact of an intervention/ programme, but also the context wherein it is implemented and associated contextual factors [30]. Thus, this framework is congruent with the notion of implementation science and commensurate to the aims of the current study. This framework measures the following five dimensions: (1) the reach into the target population, (2) the potential effectiveness of the intervention, (3) adoption by staff and the setting, (4) implementation fidelity, and (5) implementation maintenance (i.e. the degree to which the programme is sustained over time).

## Methods/design

This protocol paper has been reported in accordance with extension guidelines to the Consolidated Standards for Reporting and Writing a pilot or feasibility trial [23, 31] and the standards for reporting implementation studies statement [32]. The SPIRIT statement was found in Supplementary file. The programme was approved by the Human Subjects Ethics Application Committee of the Hong Kong Polytechnic University (No. HSEARS20201130001).

### Study design

A type 1 hybrid effectiveness-implementation design will be used in this pilot study. This design takes a dual focus in testing effects of a clinical intervention on relevant outcomes while observing and gathering information on implementation [21].

### Study setting, subjects, and recruitment strategies

The study will be implemented in a community centre operated by a non-governmental organisation. The centre provides social activities to the community-dwelling older adults and support services to their informal caregivers. Members of the community centre will be screened and recruited to the study if they (1) are aged at least 60 years, (2) currently use a smartphone, and (3) score 22 or more in the Hong Kong version of the Montreal Cognitive Assessment (HK-MoCA) [33]. Elderly individuals will be excluded if they (1) unable to communicate, (2) are not living at home, (3) are bedbound, (4) are living in an area with no Internet coverage, (5) have already participated in another structured community health and social service partnership programme, or (6) will not be staying in Hong Kong for the next 3 months.

A staff in the centre who will not involve in recruitment and the project will provide a member list to the research team. A trained research assistant (RA) will screen the member list and make sure the potential subjects will fit for the inclusion and exclusion criteria before making phone calls to them. After briefly introduce the programme to potential subjects through phone call, the RA will arrange a home visit with them to fully explain the procedure of the programme and obtain their consent forms. The RA will collect the baseline data of those who agree to participate. After successfully collecting their data, the RA will call the principal investigator (PI) of the research team for group randomisation. The subjects will be randomly assigned in a 1:1 ratio to either the intervention group or control group. Random group assignments will be generated by the PI using Research Randomizer software (i.e. 1 = intervention group; 2 = control group). These group assignments will then sealed in envelopes and opened sequentially by the PI after receiving the call from the RA. The RA and the community centre staff, but not the PI or the healthcare providers, will be blinded to the group assignments. The PI will neither involve in recruitment process nor data collection. To prevent contamination between the intervention and control groups within the centre, strategies such as clearly communicate the procedures and interventions specific to each group and provide ongoing reminders throughout the study, maintain confidentiality regarding group assignments, and implement regular monitoring and supervision of the study activities will be adopted.

### Sample size

Given that this is a pilot implementation study, the sample size will follow the rule of thumb of a minimum of 30 in each group [34]. As a previous study using a health-social service partnership programme for community-dwelling older adults had an attrition rate of approximately 15% [8], we will assume a 20% drop-out rate in this study. Therefore, a sample size of 36 will be needed for each group, for a total of 72 subjects in the two groups.

### Ethical considerations

Ethical approval has been granted by the Departmental Research Committee (Department of Nursing) on behalf of the PolyU Institutional Review Board (ref. HSEARS20201130001) of Hong Kong Polytechnic University. Information about the study’s significance, purpose, procedures, risks, and benefits will be provided to all eligible subjects. Subjects will be reassured that they can refuse participation and withdraw from the study anytime without penalty. Subjects will sign a consent form after they have expressed their understanding of the study. A telephone hotline will also be provided for subjects to ask questions about the study.

### Intervention group

**Fig. 1:**
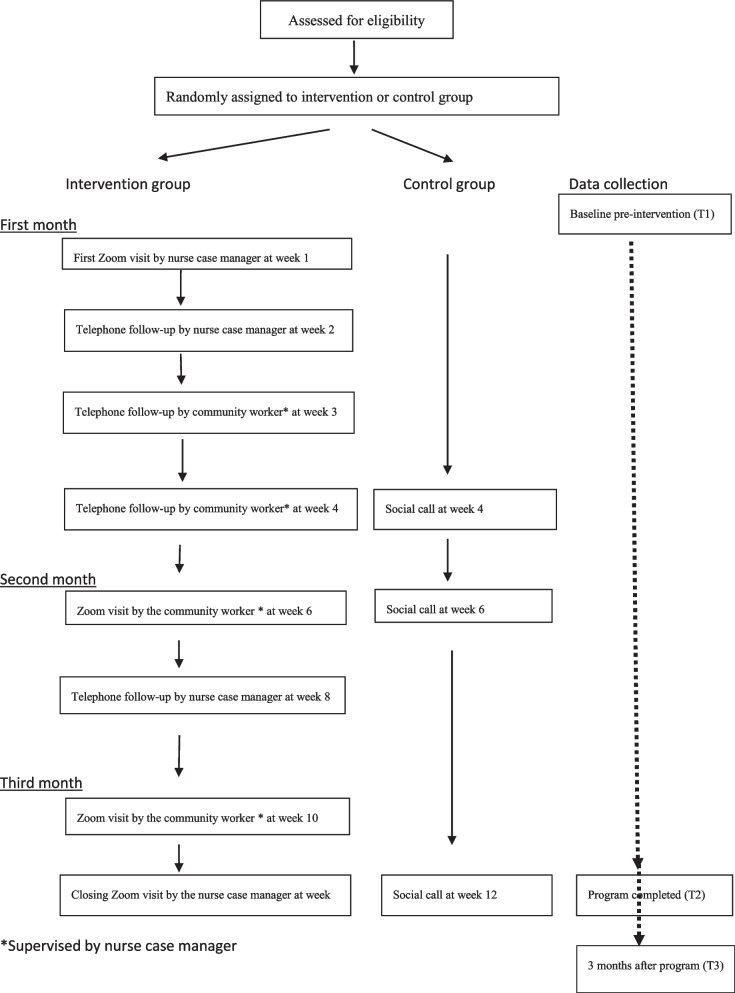
Study flow of the programme

As shown in Figure 1, each subject in the intervention group will receive four Zoom video conference sessions and four telephone calls conducted by a nurse case manager (NCM) and a community worker (CW) who will be supervised by the NCM during the 3-month programme. In the first Zoom meeting, the NCM, who has more than 20 years of community elderly care experience, will comprehensively assess the health and social problems of the subjects using the Omaha System. The Omaha System is a holistic assessment tool that evaluates environmental, physiological, psychosocial, and health-related behaviours, and covers 42 problems [35]. For example, the problems of sanitation and residence belong to the environmental domain, while the problems of respiration and cognition belong to the physiological domain. After the problems are identified, the NCM will empower the subjects to set realistic goals; identify facilitators and barriers that may promote or hinder the achievement of these goals; provide health and self-care education; and build self-efficacy by verbal encouragement, recalling previous effective self-care strategies, and taking note of the beneficial effects of adopting self-care behaviour [28]. After the first Zoom meeting, the CW will follow-up with the subjects, under the supervision of the NCM, and evaluate their progress in achieving their goals. The CW will encourage the subjects to work on their self-care goals and maintain self-care behaviour and will provide information about the health and social activities that are organised by the community centre. To reduce the risk of infection during the coronavirus (COVID-19) pandemic, Zoom meetings and telephone calls will be used as the delivery channels.

When deemed necessary, the NCM will refer the subjects to one of the health and social care team members, such as a general practitioner, traditional Chinese medicine practitioner, or social worker. The referral guidelines and protocols will be co-developed by the health and social care team. The roles and responsibilities of each member in the team will be included in the protocols with the full support of the health and social care sectors. In addition, monthly interdisciplinary care conferences will be organised to allow the subjects to discuss their progress, address their concerns, and modify and revise their care goals.

To facilitate the implementation of the programme in the community centre, the research team has held several meetings with the staff in the community centre to gain initial agreement for collaboration and to discuss the potential benefits of the programme to the community centre and the older adult members. A formal presentation was also given to inform the managers and staff at the community centre about the study rationale, timeline, roles and responsibilities of the staff, recruitment process, and referral criteria. The research team members will be informed that there will be telephone support should they encounter difficulties during the study process.

The research team has gathered feedback from the centre managers and staff on the logistics of the programme and from older adults on their preferences so that the programme can be integrated into one of the existing services provided by the community centre. To prevent research design failure, the research team will provide training to all key stakeholders, including health and social care providers and community centre managers and staff.

Before the commencement of the programme, the research team will organise a 3-day training workshop for the NCM and the centre service providers, such as the administrative staff, social workers, and CWs. The training content will include the implementation process, theoretical knowledge and practical skills, documentation processes, and the referral guidelines and support system. Didactic lectures and role playing will be used to ensure that the service team members understand and are able to apply what they have learned in a case scenario. To ensure that the centre staff are competent at applying what they have learned in the programme, the NCM will use the Omaha System to conduct a comprehensive assessment, identify any problems, and provide interventions in a simulated case according to the validated protocols. In addition, the centre staff will be required to complete and pass a post-training competence test to confirm that they have mastered the knowledge required for the subjects’ recruitment procedure.

Appropriate policies and sufficient funding are important for sustaining a programme in the community. This programme will not have the capacity to change policy, but the team will capitalise on the opportunities provided in the current policy directives to work with the social service sector to promote health in the community. In recent years, the Hong Kong government has embraced the importance of primary healthcare services and has provided support and funding to non-governmental organisations to address the health and social needs of citizens.

### Control group

Subjects in the control group will receive a monthly social telephone call from a trained research assistant. The purpose of the call will be to control the possible social effects (that is, a person will have a better social health when someone is regularly calling or caring about the person) of the intervention. When subjects have concerns about their health or have social problems, the RA will suggest that the subjects seek help from their general practitioner.

### Outcome measures

RE-AIM [30] will be adopted as the evaluation framework of the programme. Of the five dimensions included in the RE-AIM framework, only effectiveness and maintenance outcomes will be collected from both the intervention and control groups. The outcomes of the other three dimensions—reach, adoption, and implementation—will only be collected from subjects in the intervention group.

To determine whether the programme reaches the target population, the recruitment rate and subject characteristics will be collected. The recruitment rate will be calculated by dividing the total number of subjects recruited by the total number of eligible subjects in the member list of the community centre.

Since this is a pilot study, we can only measure the effectiveness outcomes if such data can be collected. The Chinese version of the General Self-Efficacy Scale will be used to measure self-efficacy level. This scale has been validated in a Chinese population and has been shown to have good validity and reliability [36]. Version 2 of the 12-item Short Form Health Survey will be used to measure quality of life [37]. This scale measures various aspects of physical and mental health, from which physical and mental component scores will be calculated. It has been widely used, with good validity and reliability [37]. The total number of unscheduled general out-patient department, general practitioner, and emergency room visits and hospital admissions and the total number of attendances at health services will comprise the health service utilisation outcomes in this study. Health service utilisation information will be collected by the subjective reporting of the subjects and will be confirmed by examining the medical and attendance certificates. This data collection protocol has been shown to have good reliability [8].

We will explore the programme adoption by using semi-structured group interviews with the providers (i.e. the social workers and the NCMs), the involved centre staff members, and eight participants (i.e. 20% of the intervention group participants). In the interviews, the research assistant will explore the interviewee’s perceptions on the facilitating factors of and barriers to program adoption in the community centre. In addition, the research assistant will also audio-record and transcribe the conversation to examine the logistic and feasibility of the programme during the interdisciplinary care conferences.

The extent to which the intervention is implemented as intended will be analysed using a performance checklist that records each step of the implementation process of the study. The checklist will be designed and completed by the PI, who will not be involved in implementing the intervention.

The maintenance effects of the intervention will be measured by repeating the measurement of effectiveness outcomes at three months following the completion of the intervention if such data can be collected.

### Data collection

Quantitative data will be collected at baseline, before the commencement of the programme (T1); immediately post-intervention (T2); and 3 months after the completion of the intervention, to evaluate the maintenance of the programme (T3). A trained RA who is blinded to the group assignment will call the subjects to collect these data.

Three separate semi-structured interviews will be conducted at T2 to collect qualitative data from all centre staff and service providers (i.e. nurse case managers and social workers) and 20% of the intervention group subjects (i.e. 8 subjects).

### Data analysis

Data will be analysed using Statistical Package for Social Sciences version 26 software (SPSS Inc., Chicago, IL, USA). Descriptive statistics will be calculated, and the distributions of the continuous variables will be examined for normality and homogeneity of variance.

Independent *t*-test will be used to analyse the differences between two groups, while repeated measures ANOVA will be adopted to measure the within-group differences. Intention-to-treat analysis will be used as the primary analysis method. Intervention effects will be reported as model-based means (95% confidence interval [95% CI]) and a *p*-value (level of significance). A significant result will be indicated by a *p*-value of less than .05 for a two-tailed test.

The principles of thematic analysis will be used in a deductive manner to analyse the qualitative data collected in the semi-structured interviews. One of the research team members will audio-record and transcribe the interviews for the team to examine the raw text and identify relevant themes. The team will discuss the text and construct a framework for analysis using codes and categories. All discrepancies will be resolved by consensus. The study will employ the framework that was proposed by Lincoln and Guba to ensure trustworthiness [38]. This framework encompasses four constructs: credibility, transferability, dependability, and confirmability. To establish credibility, we will utilise probes, prompts, and an iterative mode of questioning during the interviews. To enhance transferability, we will provide a comprehensive description of the study processes and emerging findings, enabling readers to assess the applicability of the findings to their own contexts. To ensure dependability, detailed documentation of the entire research process will be maintained and emphasised in the manuscript. To achieve confirmability, only participants who meet the eligibility criteria will be included in the interviews. The research team engage in discussions with subjects to validate the interpretations of the study findings. Additionally, involving native speakers who are also fluent in English and seeking ongoing consultation with the wider research team contribute to the rigorous execution of the study.

## Discussion

This paper describes a study protocol for the implementation and evaluation of an evidenced-based, nurse-led HSPP in a community setting. The intervention has already been shown to be effective at improving self-care in community-dwelling older adults. To fully realise the public health benefits of the intervention, attention needs to be devoted to implementation and dissemination, because of the multilevel nature of the successful integration of the programme within health and social care practice, as illustrated by Bronfenbrenner’s ecological systems theory [27].

Lorig’s Chronic Disease Self-Management Programme is one of the few nationally disseminated evidenced-based programmes that followed the traditional research pipeline from feasibility to efficacy to implementation studies [39, 40]. However, the implementation facilitators and barriers were only addressed during the national dissemination phase, which greatly restricted its potential to be disseminated in other regions [41]. The current study protocol uses a hybrid design to address the implementation concerns raised by Lorig’s national programme, including considering organisational readiness for implementation, in terms of managerial and administrative support of the community centre, at the pilot stage, to formulate more effective implementation strategies for future large-scale studies.

Glasgow and Emmons [42] identified the following four categories of barriers to the translation of effective interventions into practice: the intervention, the target setting, the research or evaluation design, and the interactions between the former three categories. To fulfil the complex self-care needs of older adults, relevant intervention programmes are necessarily intensive and demanding for community centre staff and service providers. Considering that the implementation science approach moves beyond “does it work” to “what works and for whom”, findings from this study have the potential to help with the identification of the minimal effective dose of the programme to alleviate the workload of the providers and increase their motivation to adhere to the intervention protocols [43]. In addition, the capacity of community centres to adopt additional health promotion intervention programmes is often restricted, because the staff face competing demands in providing multiple diverse services for older adults [44]. The present study aims to increase the capacity of community centres to incorporate the programme by using the prevention delivery system of Durlak and DuPre’s determinant framework. This system includes listening to and addressing the concerns of both the centre staff and service providers and reducing the complexity of the programme logistics. As a part of the implementation process, the research team will provide training to all key stakeholders, including health and social care providers and community centre managers and staff [45]. Additionally, meetings and discussions are done in advance to prevent inconsistency between the setting and the centre’s usual practice.

Despite the long-established merits of integrating health and social partnerships in international public health services, as evidenced in the literature and by political momentum, implementation challenges remain and have impeded the adoption of such partnerships [46]. In fact, a lack of communication between the health and social service sectors is considered to be a major obstacle to planning and delivering integrated care services to older adults [47]. To develop effective partnerships and allow health and social care providers to understand their roles and responsibilities in the team, this study will include training sessions before the commencement of the programme and regular team meetings to discuss the needs of both the team and the older adults.

The research team has anticipated several limitations of this study. First, the programme will only be implemented in a single community centre, and therefore, the findings may not be generalisable to other populations or other community settings. However, after identifying the facilitators and barriers and the optimal implementation strategies in this pilot study, the programme will be adopted in six community centres to further investigate its effectiveness. Second, the staff turnover rate at community centres is high. The research team will organise regular training workshops for new staff to maintain the fidelity of the programme.

## Conclusion

A nurse-led HSPP aimed at improving self-management in community-dwelling older adults was proven to be effective in a tightly controlled randomised trial. This pilot study will use a hybrid effectiveness-implementation design to collect information regarding the intervention effects and the facilitators of and barriers to the implementation of the intervention. The results may enable this programme to be implemented and sustained in the real-life service context in the community. If shown to be effective, the programme assessed may have a higher probability of being sustained in a real community setting, as it will be built with the engagement of the service partners.

### Supplementary Information

**Additional file 1.**

## Data Availability

The datasets used and/or analysed during the current study are available from the corresponding author on reasonable request.
